# Safety and immunogenicity of four-segmented Rift Valley fever virus in the common marmoset

**DOI:** 10.1038/s41541-022-00476-y

**Published:** 2022-05-18

**Authors:** Paul J. Wichgers Schreur, Petra Mooij, Gerrit Koopman, Babs E. Verstrepen, Zahra Fagrouch, Daniella Mortier, Nikki van Driel, Jet Kant, Sandra van de Water, Willy M. Bogers, Carine Punt, Lucien van Keulen, Ernst J. Verschoor, Jeroen Kortekaas

**Affiliations:** 1 Department of Virology and Molecular Biology, Wageningen Bioveterinary Research, Wageningen University & Research, Lelystad, The Netherlands; 2 BunyaVax B.V., Lelystad, The Netherlands; 3 Department of Virology, Biomedical Primate Research Centre, Rijswijk, The Netherlands; 4 Laboratory of Virology, Wageningen University & Research, Wageningen, The Netherlands

**Keywords:** Live attenuated vaccines, Viral infection

## Abstract

Rift Valley fever virus (RVFV) is an emerging mosquito-borne bunyavirus that is highly pathogenic to wild and domesticated ruminants, camelids, and humans. While animals are exclusively infected via mosquito bites, humans can also be infected via contact with contaminated tissues or blood. No human vaccine is available and commercialized veterinary vaccines do not optimally combine efficacy with safety. We previously reported the development of two novel live-attenuated RVF vaccines, created by splitting the M genome segment and deleting the major virulence determinant NSs. The vaccine candidates, referred to as the veterinary vaccine vRVFV-4s and the human vaccine hRVFV-4s, were shown to induce protective immunity in multiple species after a single vaccination. Anticipating accidental exposure of humans to the veterinary vaccine and the application of hRVFV-4s to humans, the safety of each vaccine was evaluated in the most susceptible nonhuman primate model, the common marmoset (*Callithrix jacchus*). Marmosets were inoculated with high doses of each vaccine and were monitored for clinical signs as well as for vaccine virus dissemination, shedding, and spreading to the environment. To accurately assess the attenuation of both vaccine viruses, separate groups of marmosets were inoculated with the parent wild-type RVFV strains. Both wild-type strains induced high viremia and disseminated to primary target organs, associated with mild-to-severe morbidity. In contrast, both vaccines were well tolerated with no evidence of dissemination and shedding while inducing potent neutralizing antibody responses. The results of the studies support the unprecedented safety profile of both vaccines for animals and humans.

## Introduction

Rift Valley fever virus (RVFV) belongs to the order *Bunyavirales* (genus *Phlebovirus*, family *Phenuiviridae*) and is the causative agent of Rift Valley fever (RVF). The mosquito-borne virus affects wild and domesticated ruminants, such as sheep, goats, cattle, buffalo, and camelids, thereby presenting with liver necrosis and hemorrhagic manifestations, with fatal outcomes especially in young animals. Outbreaks are characterized by abortion storms in sheep herds. In endemic areas, epizootics are generally associated with explosive mosquito populations following heavy rains. A wide range of mosquito species have been associated with transmission of the virus, particularly those within the genera *Aedes* and *Culex*^1,2^. The virus is currently widespread in Africa and is expected to expand its habitat further, stimulated by climate change and globalization^3^.

In addition to infecting ruminants, RVFV is also infectious to humans. Humans can be exposed to the virus via contact with contaminated tissues or blood released during the slaughtering of RVFV-infected animals, or via mosquito bites. Although humans generally develop a self-limiting febrile illness, a significant fraction develops neurological disorders or hemorrhagic fever^4^, which is often fatal. Furthermore, increasing evidence suggests that RVFV infection during human pregnancies may result in complications^5–7^. Presently there are no vaccines or therapeutics available to prevent or treat human infections.

The RVFV genome is divided into three RNA segments of negative polarity that are named after their size; small (S), medium (M), and large (L). The L segment encodes the viral RNA-dependent RNA polymerase. The S segment encodes the nucleocapsid that protects the viral RNA from degradation and a non-structural protein named NSs. NSs interferes with host innate immune signaling pathways and is considered the major virulence determinant of the virus^8^. The M segment encodes a polyprotein precursor that is co-translationally cleaved by host proteases into the structural glycoproteins Gn and Gc. Gn is involved in attachment to target cells and Gc is required for the fusion of the viral and endosomal membranes. The M segment additionally encodes a small 14-kDa protein, named NSm, which counteracts apoptosis^9^, and a large 78-kDa glycoprotein, named LGp, that comprises the NSm and Gn coding regions, shown to be important for the dissemination of the virus in mosquitoes^10–12^.

We previously reported the development of a novel live-attenuated RVF vaccine that was constructed by splitting the M genome segment into two M-type segments, one encoding Gn and the accessory proteins NSm and LGp, and one encoding Gc, resulting in a four-segmented RVFV (RVFV-4s)^13^. To optimize the safety profile, the NSs gene was deleted from the S segment as well. The technology to create four-segmented RVF viruses was subsequently used to create tailor-made vaccines for both animal and human applications. The veterinary vaccine, here referred to as “v”RVFV-4s, is based on strain 35/74, isolated from the liver of a sheep that died during an RVFV outbreak in South Africa in 1974^14^, whereas the vaccine to be applied in humans, here referred to as “h”RVFV-4s, is based on the Clone 13 strain that is derived from the pathogenic 74HB59 strain isolated from a human case in the Central African Republic^15,16^. The vRVFV-4s strain lacks the NSs gene and the hRVFV-4s strain contains the S genome segment of Clone 13 with a 69% deletion of its NSs gene. Strain vRVFV-4s optimally replicates in BHK-21 and BSR-T7 cells, both suitable for veterinary vaccine production, whereas hRVFV-4s replicates optimally in Vero cells, which are frequently used for human vaccine production (Supplementary Fig. 1)

Both vRVFV-4s and hRVFV-4s were shown to be safe and efficacious in various rodent and ruminant animal models, including a reversion to virulence study, performed according to the recommendations of the OIE Terrestrial Manual Chapter 2.1.18 and the Ph. Eur. 5.2.7 monograph^17^. However, safety data obtained with a more appropriate model for humans was lacking. Demonstration of safety in a nonhuman primate (NHP) model is not only relevant for the candidate human vaccine, but also for the candidate veterinary vaccine, as veterinarians applying this vaccine could be exposed accidentally to the vaccine virus. NHPs, considering their close phylogenetic relationship to humans, are one of the most predictive models regarding human vaccine safety and efficacy^18^. Historically, rhesus macaques were used for the evaluation of candidate RVF vaccines and therapeutics, despite their relatively low susceptibility to the wild-type virus^19,20^. More recently, common marmosets (*Callithrix jacchus*) were shown to provide a more convenient model, as these animals develop higher morbidity, more consistent viremia, and marked aberrations in hematological and biochemistry values following wild-type RVFV infection compared to macaques^21,22^.

In this study, we evaluated the safety and immunogenicity of a high dose of the vRVFV-4s vaccine candidate and of three different doses of the hRVFV-4s vaccine candidate in common marmosets. We specifically assessed body temperatures, virus dissemination and shedding, and induction of neutralizing antibody responses compared to their parent wild-type RVFV strains.

## Results

### Experimental design

**Fig. 1:**
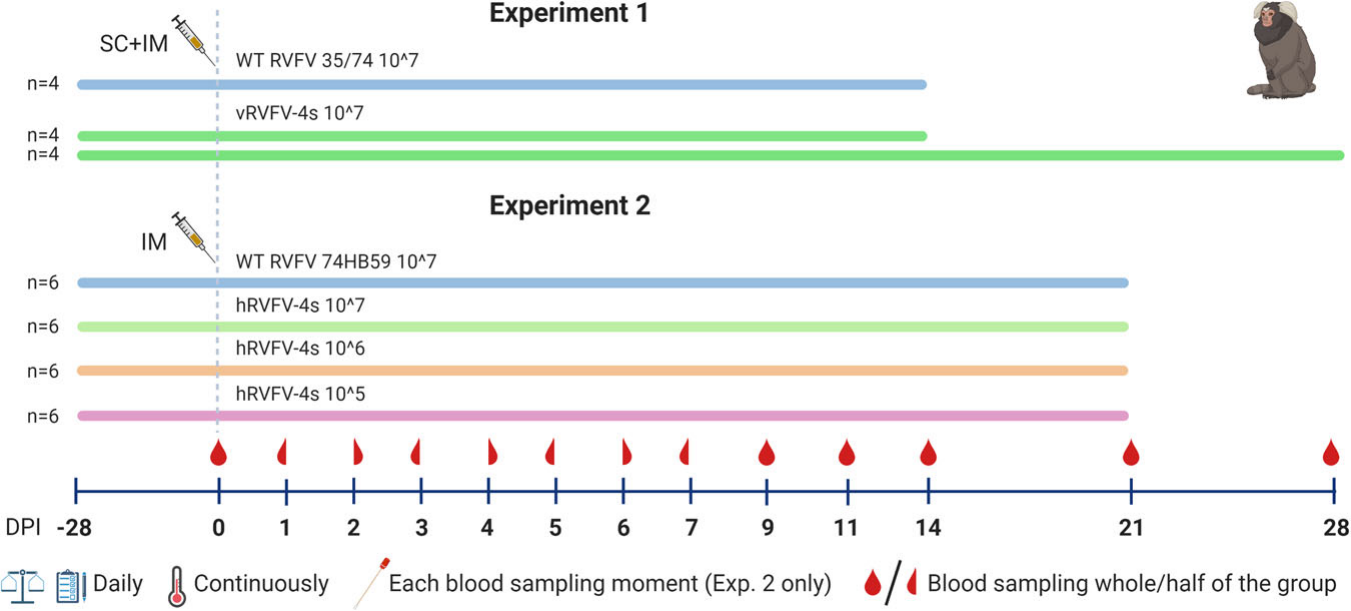
Schematic representation of the experimental design. Marmosets of experiment 1 were inoculated with vRVFV-4s and in experiment 2 with hRVFV-4s with the indicated dose and route. In both experiments, control animals were inoculated with the wild-type parent strains; for vRVFV-4s this is strain 35/74 and for hRVFV-4s this is 74HB59. Blood samples were taken regularly as indicated, and body temperatures were monitored continuously by an abdominal implanted AniPillV2. Oral and rectal swabs were taken only during experiment 2.

To assess the safety and immunogenicity of both vRVFV-4s and hRVFV-4s in the marmoset model, we conducted two independent experiments (Fig. 1). In experiment 1, eight marmosets were inoculated with a dose of 10^7^ TCID_50_ of vRVFV-4s via a combined intramuscular (IM) and subcutaneous (SC) route. The combined (IM + SC) administration route was chosen as preferred vaccination route because at the start of the study no optimal vaccination route for ruminants was yet defined. On day 14 and day 28, four inoculated animals were euthanized to collect organ samples. Another four marmosets were inoculated with a 10^7^ TCID_50_ dose of parent strain 35/74 and were necropsied on day 14. In experiment 2, three groups of six animals were inoculated with either 10^5^, 10^6^, or 10^7^ TCID_50_ of hRVFV-4s via IM route only, as the IM route is the anticipated route for human vaccination. In this experiment, a group of identical size was inoculated with 10^7^ TCID_50_ of virulent parent strain 74HB59. At 3 weeks post inoculation, all animals of experiment 2 were euthanized and the organs were collected. Animals in both experiments were frequently monitored for body weight and clinical signs, besides continuous monitoring of body temperatures. Furthermore, blood samples were regularly collected for hematological, biochemical, virological, and immunological analyses. To assess potential virus shedding, oral and rectal swabs were taken in experiment 2.

### RVFV-4s inoculated animals do not exhibit behavior changes, clinical signs, or weight loss

**Fig. 2:**
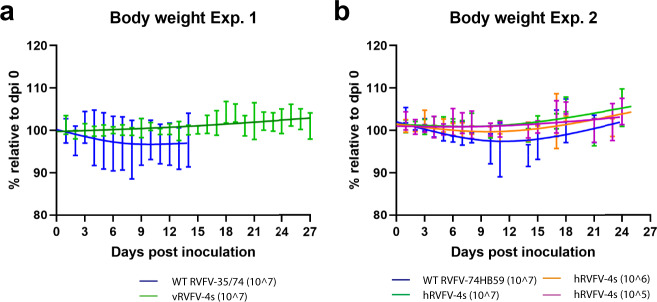
RVFV-4s inoculated animals demonstrate no significant weight loss. Body weights relatively to day post inoculation (DPI) 0 measured in experiment 1 (**a**) and experiment 2 (**b**). Standard deviations are presented at each time point and spline trendlines were added (GraphPad Prism 9.0). No statistically significant differences were observed between groups.

None of the animals inoculated with vRVFV-4s (experiment 1) or hRVFV-4s (experiment 2) presented abnormal behavior or manifested with clinical signs or weight loss during the experiment (Fig. 2a, b). One out of four wild-type RVFV 35/74 inoculated animals (experiment 1, animal M12030) showed tremors starting on day 4 post inoculation. These clinical signs worsened on day 15 and the animal was euthanized consequently. This animal additionally presented with substantial weight loss following inoculation. Of note, this animal presented with some (unaccountable) weight loss already prior inoculation. The other three animals in this group did not show clinical signs and were euthanized at the end of the experiment. In experiment 2, approximately 11 days post inoculation (DPI), four out of six control animals inoculated with wild-type strain 74HB59 (M16010, M16017, M16040, and M17011) started to show clinical signs, such as unkempt coats and hunched postures. The general health of animals M16010 and M17011 gradually improved over time, but at 13 and 14 DPI the physical condition of marmosets M16040 and M16017 deteriorated to a lethargic state, reaching a pre-defined humane endpoint. Of note, animal M17058 presented with shallow breathing, unkempt coat, and a hunched posture for 2 days starting on day 14 post inoculation. This animal recovered but maintained shallow breathing until the end of the study (day 21). Disease severity was more pronounced in marmosets inoculated with the 74HB59 strain compared to strain 35/74. No behavioral changes, clinical signs, or weight loss were observed in any of the vRVFV-4s or hRVFV-4s inoculated animals.

### Transient, dose-dependent body temperature increase in RVFV-4s inoculated marmosets

**Fig. 3:**
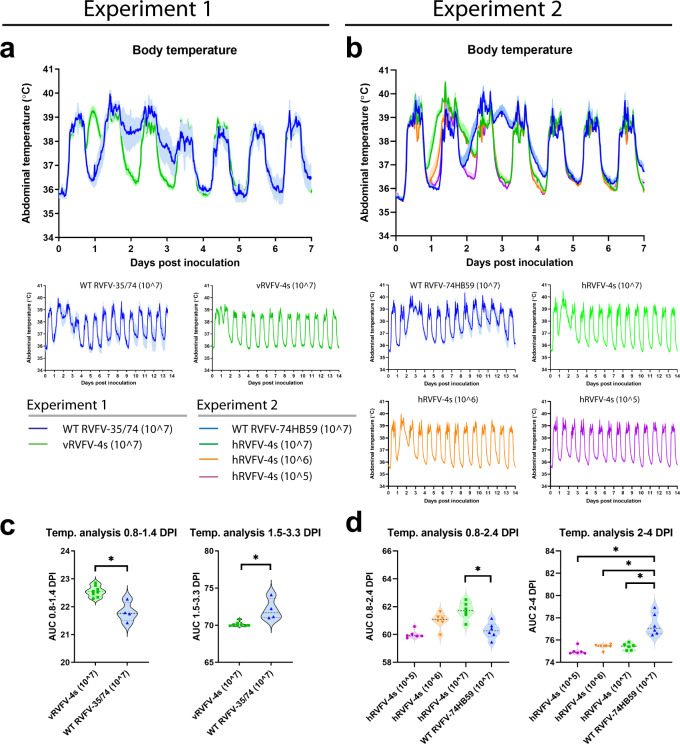
Dose-dependent early-phase temperature increase following inoculation of marmosets with RVFV-4s. Body temperatures in experiment 1 (**a**) and experiment 2 (**b**) were recorded every 15 min using an AniPill telemetric device in the abdominal cavity. Averages and SEM are presented for each group. Area under the curve (AUC) measurements were performed at the indicated days post inoculation (DPI) to assess statistically significant differences between groups of experiment 1 (**c**) and, groups of experiment 2 (**d**). Data are presented as violin plots with medians (dashed line) and were analyzed by (**c**) unpaired *t*-test or (**d**) one-way ANOVA using Dunnett’s multiple comparisons test and comparing the individual vaccine groups to the corresponding wild-type virus groups (GraphPad Prism 9.0). Statistical significance (*p* value ≤ 0.05) is indicated with an asterisk (*).

All animals were implanted with an AniPill, a telemetric device to continuously monitor the abdominal body temperatures. Before inoculation, all animals showed a normal circadian pattern with a body temperature of approximately 39 °C during the day versus 36 °C during the night. Following inoculation with either vRVFV-4s or hRVFV-4s, a consistent transient elevation of the night-time body temperatures was observed in all animals that received a 10^7^ TCID_50_ dose, starting at DPI 1, and persisting for approximately 24 h (Fig. 3). At lower doses (10^6^ or 10^5^ TCID_50_, experiment 2), the transient elevation was less pronounced. Marmosets inoculated with the wild-type virus did not show this early-phase temperature increase but instead had a more pronounced temperature increase at 2–4 DPI that persisted on average for 1.5–2 days (Fig. 3).

### RVFV-4s inoculation results in minor changes in hematology and no clinical chemistry changes

**Fig. 4:**
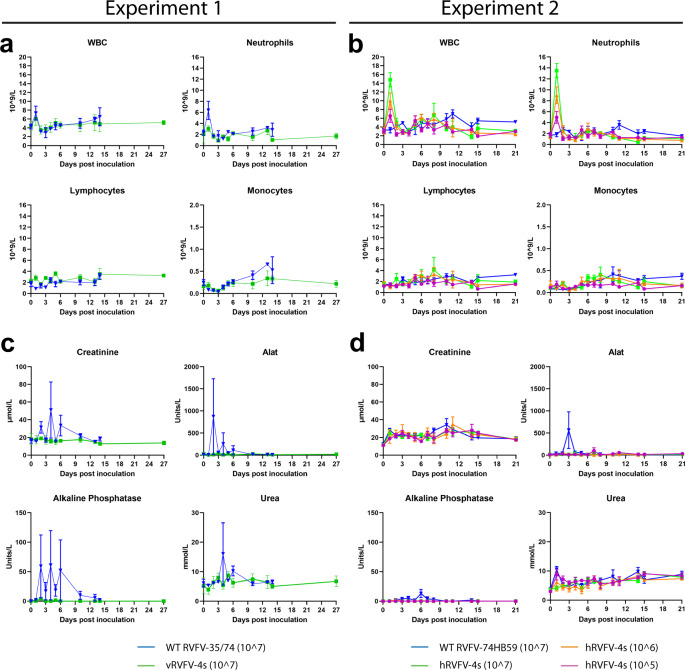
Short-term neutrophilia following hRVFV-4s inoculation and no changes in blood chemistry following either vRVFV-4s or hRVFV-4s inoculation. WBC, neutrophil, lymphocyte, and monocyte counts measured in blood collected from animals from experiment 1 (**a**) and animals from experiment 2 (**b**) at the indicated timepoints. Creatinine, alanine aminotransferase (ALAT), alkaline phosphatase (ALP), and urea levels in plasma measured in animals from experiment 1 (**c**) and animals from experiment 2 (**d**), at the indicated timepoints. In the first week post inoculation, blood samples were obtained every other day from an individual animal (Fig. 1). Averages and SEM are presented for each group. No statistical analysis could be performed due to the low number of measurements per time point.

Following inoculation with wild-type strain 35/74 in experiment 1, both animals sampled on day 1 post inoculation (M15045 and M15046) showed a minor increase in white blood cell (WBC) and neutrophil counts (Fig. 4). No changes were observed at other timepoints and no changes were observed in lymphocyte and monocyte counts. In the vRVFV-4s inoculated animals all cell numbers remained within the normal range as observed with historic control animals at all timepoints assessed. In experiment 2, the overall hematology pattern slightly differed compared to experiment 1. The WBC and neutrophil counts on day 1 post inoculation (three out of three animals assessed) were elevated compared to day 0 following hRVFV-4s inoculation. The animals inoculated with 10^7^ TCID_50_ had higher WBC and neutrophil counts compared to the groups inoculated with lower doses, suggesting a dose response. No increases in WBC and neutrophil counts were observed in animals inoculated with the corresponding wild-type strain. Furthermore, no differences were observed in lymphocyte or monocyte counts following inoculation of either wild-type strain 74HB59 or hRVFV-4s.

As RVFV is a hepatotropic virus we also assessed the levels of systemic liver enzymes and renal function parameters in both experiments. Following inoculation with the wild-type strains (experiments 1 and 2), no major changes were observed, except for animal M12030 that presented with increased liver enzymes and renal function parameters in plasma (Fig. 4). No changes in the blood chemistry of the vRVFV-4s or hRVFV-4s inoculated animals were observed.

### Absence of viremia in RVFV-4s inoculated animals

**Fig. 5:**
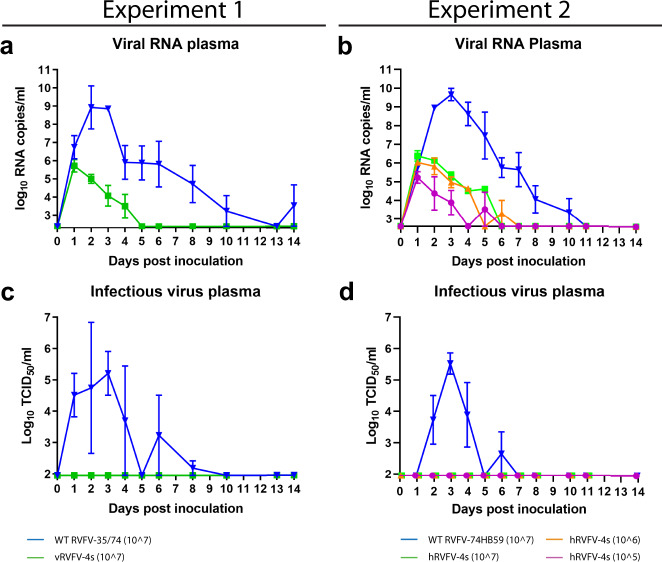
No infectious virus recovered from plasma of RVFV-4s inoculated marmosets. Plasma samples were assessed for the presence of viral RNA by RT-qPCR (**a**, **b**) and for infectious virus by virus isolation (**c**, **d**). Samples that tested negative are depicted at the detection limit of the PCR (2.6 log10 RNA copies/ml) or virus isolation (1.95 log10 TCID_50_/ml). In the first week post inoculation, the animals were allotted to one of two sampling groups. Animals in each group were bled every other day to minimize withdrawal volumes (see Fig. 1). Averages and SEM are presented for each group.

One of the key safety parameters for live-attenuated vaccines is absent or limited vaccine virus viremia in the vaccinated individual, thereby reducing or even preventing the unwanted introduction of the vaccine into the environment, either through shedding or via mosquitoes. To investigate if RVFV-4s induces viremia in marmosets, plasma samples were collected at regular timepoints after inoculation and tested for the presence of viral RNA and infectious virus. In both experiments, high levels of viral RNA and infectious virus were detected in plasma of marmosets inoculated with the parent viruses at 2–4 DPI. Both viral RNA and infectious virus gradually decreased to undetectable levels at 11 and 7 DPI, respectively (Fig. 5a–d). Peak viremia in animals inoculated with wild-type virus coincided with increases in abdominal body temperatures (Fig. 3). In the vRVFV-4s and hRVFV-4s inoculated animals viral RNA was detected at DPI 1 and gradually declined to a level below the detection limit around DPI 5. The total genome copy numbers detected at this time point (<10^7^/ml) were approximately three logs lower than the total number of genome copies present in the inoculum (>10^10^/ml). No increases in viral RNA levels were detected in these animals, suggesting that RVFV-4s inoculation does not result in vaccine virus viremia. This is supported by the lack of detectable infectious vaccine virus in plasma collected from RVFV-4s inoculated animals (Fig. 5c, d).

### No evidence of hRVFV-4s dissemination to RVFV target organs

**Fig. 6:**
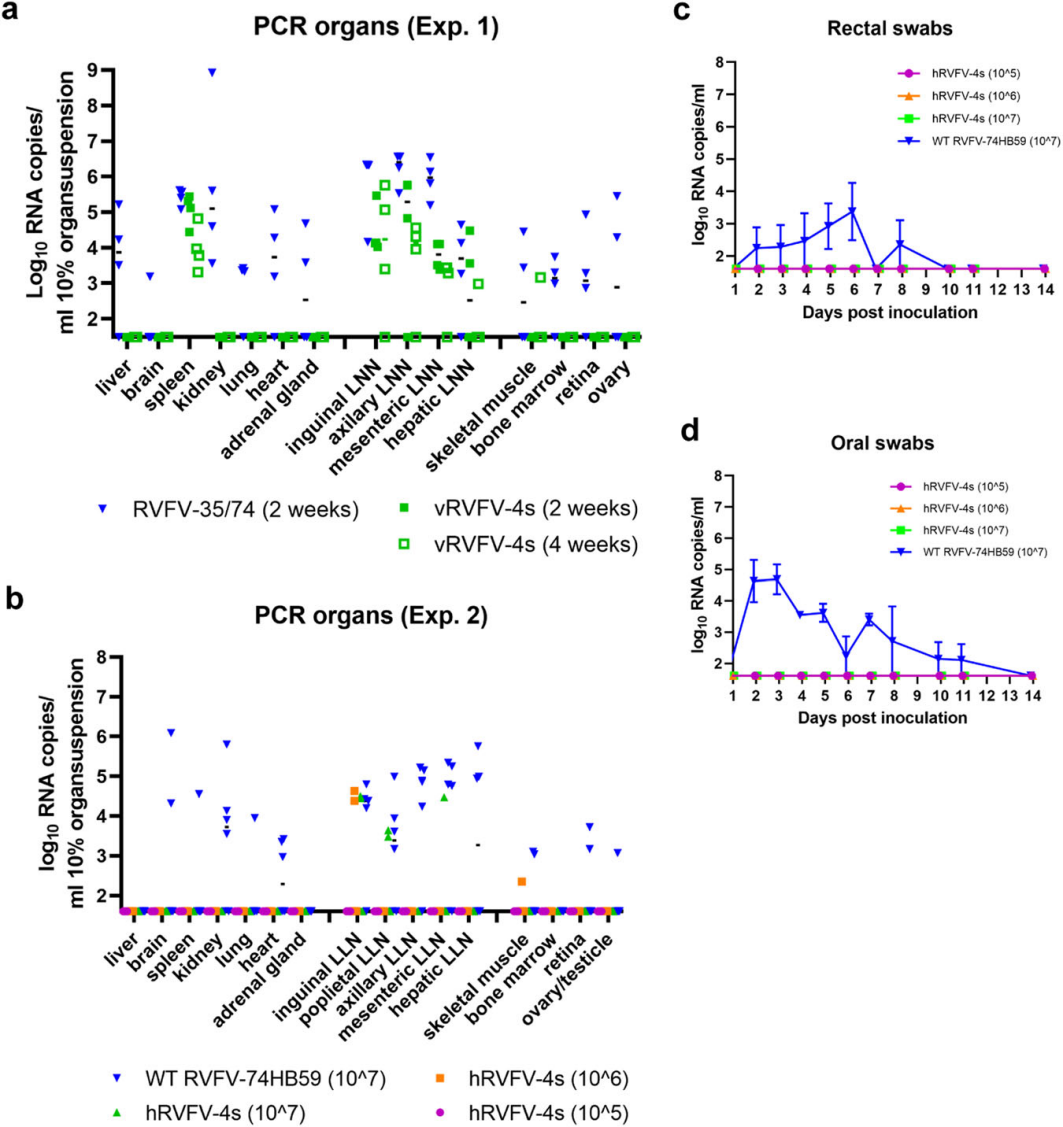
RVFV-4s does not disseminate to RVFV target organs, while the virus is most likely carried by phagocytes to lymphoid organs. Organ suspensions of marmosets from experiment 1 (**a**) and experiment 2 (**b**) as well as rectal (**c**) and oral swabs (**d**) of animals from experiment 2 were assessed for the presence of viral RNA by RT-qPCR. The *x*-axes cross the *y*-axes at the limit of detection (1.48 log10 RNA copies/ml for experiment 1 and 1.6 log10 RNA copies/ml for experiment 2). **c**, **d** Averages and SEM are presented for each group.

To determine if RVFV-4s vaccine viruses disseminated in the inoculated marmosets, we assessed a broad range of organs for the presence of viral RNA. Of note, most of the organs were obtained at necropsy at 2, 3, or 4 weeks post inoculation at which time viremia of the parent viruses had subsided. Nevertheless, in animals inoculated with the parent viruses (experiments 1 and 2) viral RNA was detected in liver, brain, spleen, kidney, lymph node, heart, and adrenal gland samples (Fig. 6a, b). Furthermore, a few bone marrow, retina, and ovary samples were positive for viral RNA, albeit at a very low level. Notably, from the animals that were euthanized upon reaching a humane endpoint, a very high PCR signal was obtained from a kidney sample of animal M12030 (experiment 1) and a moderate signal for brain samples of animals M16017 and M16040 (experiment 2). Histopathological and immunohistochemical examination revealed an extensive tubulo-interstitial nephritis in animal M12030 with strong RVFV immunolabelling of the renal tubular epithelium and proteinaceous/cellular casts within the tubuli (Supplementary Fig. 2a, b). Animals M16017 and M16040 showed a multifocal meningo-encephalitis with immunostaining of neurons and neuronal processes throughout the brain (Supplementary Fig. 2c, d).

In the vRVFV-4s inoculated animals no viral RNA was detected in liver, spleen, kidney, heart, and adrenal gland samples, but viral RNA was detected in various lymph node samples and occasionally in spleen samples. Remarkably, the overall number of RVFV-4s RNA-positive lymph node samples was higher in experiment 1 compared to experiment 2.

### Absence of hRVFV-4s shedding to the environment

To investigate vaccine virus shedding to the environment, rectal and oral swabs were obtained at various timepoints from all animals that were part of experiment 2 (Fig. 1). No swab samples were collected during experiment 1. PCR analysis revealed low levels of viral RNA in both oral and rectal swab samples collected from animals inoculated with the wild-type viruses, whereas no viral RNA was detected in any sample collected from hRVFV-4s inoculated animals (Fig. 6c, d). These results suggest that hRVFV-4s does not sheds to the environment.

### Induction of a robust immune response

**Fig. 7:**
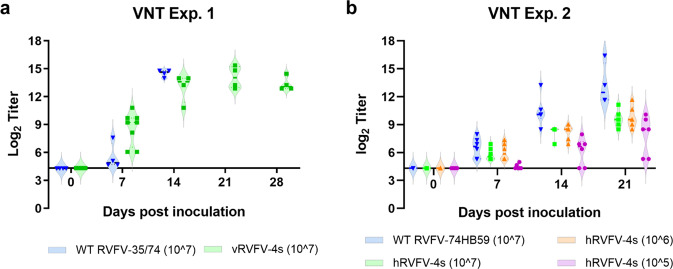
RVFV-4s inoculation results in the induction of a robust neutralizing antibody response in marmosets. Plasma samples of experiment 1 (**a**) and experiment 2 (**b**) were assessed for the presence of RVFV-specific neutralization antibodies by VNT assay and expressed as neutralization titer 50%. Violin plots (with medians) are presented with *x*-axes crossing the *y*-axes at the limit of detection.

As neutralizing antibodies are the only known correlate of protection for RVFV, we assessed those responses in both experiments. Starting from DPI 7, neutralizing antibodies were detected in both vRVFV-4s and hRVFV-4s inoculated animals, and neutralizing antibody levels increased up to 3 weeks post inoculation (Fig. 7). In experiment 2, in which several RVFV-4s doses were assessed, lower levels of neutralizing antibodies were observed in animals inoculated with 10^5^ TCID_50_, relative to the higher dose groups. No differences were observed between animals vaccinated with 10^6^ and 10^7^ TCID_50_. As expected, all parent virus-inoculated animals developed high levels of neutralizing antibodies. Remarkably, the overall levels of neutralizing antibodies of the parent virus-inoculated animals were only slightly higher compared to those detected in the vRVFV-4s and hRVFV-4s high-dose groups.

## Discussion

In earlier work, we have demonstrated that the vRVFV-4s vaccine candidate does not disseminate in inoculated ruminants and is not shed from these animals to the environment, while inducing protective immune responses after a single vaccination^17^. Anticipating accidental exposure of humans to this veterinary vaccine, we evaluated its safety in the most susceptible NHP model, the common marmoset. In a similar animal model, we evaluated the safety of the hRVFV-4s candidate human vaccine, in preparation for a first-in-human clinical trial.

For live-attenuated vaccines, it is of utmost importance to demonstrate the absence of virulence and inability to spread to the environment. The absence of viremia in RVFV-4s inoculated marmosets highlights that the risk of spread of the vaccine virus to mosquito vectors and subsequent spread to naive hosts is negligible. This is in agreement with our previous observation of the absence of viremia in ruminants^17^. Furthermore, no virus was detected in the swab samples, as part of the sample panel of experiment 2 (hRVFV-4s). Of note, the levels of viral RNA in swabs were low for the wild-type virus, in line with the inability of RVFV to spread directly from animal to animal without the involvement of mosquito vectors^23^.

While no infectious vaccine virus could be recovered from inoculated marmosets, viral RNA was detectable during the first days after inoculation in plasma samples and in lymphoid organs at the time of necropsy. It is important to realize that about 10^10^ genome copies were administrated per individual animal (1 TCID_50_ was previously shown to be equivalent to about 1000 genome copies^17^). Taking into account the small size of a marmoset, 350–400 g with about 35 ml total blood volume, it is not surprising to detect viral RNA in the circulation early after inoculation. The decline of viral RNA levels in plasma samples at 2 DPI contrasts with the exponentially increased RNA levels detected in marmosets inoculated with wild-type virus at 2 DPI. These observations suggest limited, or even absence of RVFV-4s propagation in vivo. The presence of vaccine virus RNA in lymphoid organs at the time of necropsy can be explained by phagocytosis of vaccine virus, genomic RNA, and/or phagocytosis of infected cells by migrating cells (macrophages, dendritic cells, neutrophils). Notably, the number of lymphoid organs positive for viral RNA in experiment 1 was higher compared to experiment 2. The different inoculation routes (combined IM/SC versus IM) or the different genetic lineages of the vaccine strains used in the independent experiments may be a factor explaining this finding^24^. Furthermore, as RVFV-4s inoculated animals were necropsied several weeks post inoculation, transient replication of RVFV-4s in RVFV target organs cannot be excluded.

Whereas no untoward effects or inoculation site reactions were noted in RVFV-4s inoculated animals, a dose-dependent body temperature increase was observed consistently in these animals at 24 h post inoculation. Importantly, in both experiments, the expected temperature increase in animals inoculated with wild-type virus followed a different pattern and became apparent at 2–3 DPI, which correlated with peak viremia. Most likely, the vaccine viruses were efficiently detected by the marmoset’s innate immune system and triggered innate immune responses with associated pyrexia. It is unlikely that other components besides the virus, like media or cell components, induced these pyrogenic reactions, since the inocula containing the wild-type virus was prepared using the same cells and culture medium. Considering that the NSs protein, which is only present in the parent viruses, is known to efficiently counteract early innate immune responses^8^, we hypothesize that NSs delays the induction of an innate immune response in marmosets inoculated with wild-type RVFV. It is plausible that also other live-attenuated RVFV candidate vaccines lacking NSs induce short-lived pyrexia; however, such responses may not have been noted in earlier studies as body temperatures were not measured continuously^25^, as was done in the present study. Overall, the dose-dependent pyrexia could be considered a mild side effect of the vaccines but also suggests that the vaccines induce potent innate immune responses, likely supporting subsequent adaptive responses.

As part of the safety analysis, we also assessed blood cell counts in both experiments. In general, the overall results with respect to blood cell counts of experiment 1 and experiment 2 were similar, although we observed one striking difference. In experiment 1, increases in total WBC and neutrophil levels were observed in wild-type 35/74 inoculated animals whereas no such changes were observed in vRVFV-4s inoculated animals, while in experiment 2, no increases in WBC and neutrophil levels were observed in marmosets inoculated with the wild-type virus, whereas dose-dependent increases were observed in hRVFV-4s inoculated animals. Remarkably, increased neutrophil levels were detected on only one day. We do not have a clear explanation for the differences in neutrophil counts between the experiments, but it might be related to the differences in the genetic background of the vaccines and wild-type viruses^24^. Nevertheless, the data underscore that the hRVFV-4s vaccine is recognized efficiently by the marmoset’s innate immune system. Whether memory cellular immune responses also differ after inoculation with the different vaccines awaits further study.

Altogether, the present work emphasizes that both vRVFV-4s and hRVFV-4s have a high safety profile in the marmoset model and that both vaccine viruses induce significant immune responses following vaccination, supporting further development of both vaccines for animal and human vaccination.

## Materials and methods

### Ethical approval

The study was reviewed and approved by the Dutch “Centrale Commissie Dierproeven” (AVD5020020174224) according to Dutch law, article 10a of the “Wet op de Dierproeven”. The specific experiments were approved by the Biomedical Primate Research Centre (BPRC’s) Animal Welfare Body (IvD).

### Animals and housing

The experiments were carried out using purpose-bred P2 adult common marmosets (*Callithrix jacchus*) of >350 g. In experiment 1, 12 female animals (>2.5 years old) were enrolled whereas in experiment 2, 16 male and 8 female animals were used, also >2.5 years old. Animals were identified by a colored label on a chain and an implanted microchip. The animals underwent a full physical examination, including faecal screening for pathogens and clinical chemistry and hematology evaluation before enrollment into the study. Only healthy individuals with normal clinical chemistry and hematology values were selected. Animals were housed in pairs in hBSL-3 facilities during the study period. The animals were offered a daily diet that was optimized for marmosets at the BPRC. Enrichment was offered daily, and drinking water was available ad libitum.

### Cells and viruses

Culture media and supplements were obtained from Gibco unless indicated otherwise. BHK-21 and BSR-T7 cells were maintained in Glasgow minimum essential medium (GMEM) supplemented with 4% tryptose phosphate broth (TPB), 1% minimum essential medium nonessential amino acids (MEM NEAA), 1% antibiotic/antimycotic (a/a), and 5% fetal bovine serum (FBS), at 37 °C with 5% CO_2_. Vero WHO cells were maintained in VP-SFM supplemented with 2% L-glutamine and 0.01% Gentamycin (Sigma). A master seed virus batch of vRVFV-4s (previously referred as RVFV-LMMS_delNSs_^13,17^) was prepared by infecting BSR-T7 cells with plaque-purified seed virus, cultured in complete medium (GMEM supplemented with 5% FCS [SAFC], 4% TPB, 0.01% Gentamycin and 1% NeAA) at a multiplicity of infection (MOI) of 0.002 for 3 days^17^. A pre-seed batch of hRVFV-4s was prepared by plaque-purifying BSR-T7 rescued material on Vero WHO cells and infection of Vero WHO cells at an MOI of 0.01 for 3 days. The infection medium consisted of VP-SFM supplemented with 2% L-glutamine and 2% FBS and 1% a/a. Wild-type recRVFV-35/74 was propagated and produced following low MOI infection (0.001) of BHK-21 cells, which were grown in CO_2_-independent medium (CIM), supplemented with 5% FCS, 1% L-glutamine, and 1% (a/a)^26^. Wild-type recRVFV-74HB59 was propagated and produced in BSR-T7 cells. The infection medium consisted of GMEM, 5% FBS 4% TPB, 1% MEM NeAA and 1% a/a. Wild-type strains were handled at BSL-3 containment and vRVFV-4s and hRVFV-4s at BSL-2 containment level.

### Inoculation

Animals of experiment 1 received a combined SC (between shoulder blades) and IM (upper left leg) inoculation of 500 µl at each site with a total of 10^7^ TCID_50_ vRVFV-4s or parent virus. In experiment 2, all animals received two IM (upper legs) inoculations of 500 µl at each site (left and right leg) with a total of 10^5^, 10^6^, or 10^7^ TCID_50_ of hRVFV-4s or 10^7^ TCID_50_ of parent virus RVFV-74HB59.

### Blood sampling

Blood samples were collected every other day in the first week after inoculation. To avoid exceeding the monthly maximal amount of blood (1% of body weight) to be drawn, half of the animals were bled on days 1, 3, and 5, the other half on days 2, 4, and 6. Additional blood samples were taken as presented in Fig. 1.

### General behavior and clinical signs

The animals were monitored for clinical signs twice per day. Animal caretakers were blinded for which animal received the high, medium, or low dose of the vaccine. Because animals that received the wild-type parent viruses had to be kept separate from animals that received the vaccines, the animal caretakers were not blinded for the wild-type groups. Abnormalities were communicated immediately to the principal investigator. When an animal showed signs of disease during the day, an extra observation was scheduled in the evening. The following clinical parameters were monitored: Appetite, stool consistency, general behavior, fever (measured via a telemetric device), anorexia, dehydration, vomiting, drooling, ataxia, horizontal nystagmus, head-pressing, tremors, shallow breathing, blood in urine, foaming at the mouth, seizures, activity/lethargy, hunched posture in combination with ruffled/unkept fur. These clinical signs were described previously in NHPs that succumbed after exposure to RVFV^22^.

### Body weights and temperatures

Animals were captured in a perspex cylinder and weighed as such. A telemetric device (AnipillV2 telemetry system (BodyCAP, Hérouville Saint Clair, France) was surgically placed in the abdominal cavity at 4 weeks before inoculation. This device measures the body temperature every 15 min and transfers data to a receiver enabling monitoring of fever development in real-time.

### Isolation and cryopreservation of serum and EDTA plasma

To collect blood samples, animals were sedated with Alfaxan (12 mg/kg, IM route). Blood was drawn from the femoral vein in the groin using aseptic techniques using a Vacutainer blood collection system (Becton Dickinson, Vacutainer systems). EDTA tubes with collected blood were centrifuged for 10 min at 1000 × *g* followed by the collection of plasma. A volume of 125 µl plasma was used for clinical chemistry measurements. The remaining material was stored at −80 °C for virus detection via PCR and for the detection of RVFV antibodies. Upon euthanasia, 2 ml EDTA blood and plain blood samples were collected. Serum samples were prepared by centrifugation of clotted blood for 10 min (1000–1300 × *g*), and serum was stored at −20 °C.

### Clinical chemistry and hematology

At indicated timepoints, hematology and clinical chemistry levels were measured. Hematology parameters were measured in replenished EDTA blood with a Sysmex XT-2000iV Automated Hematology Analyzer (Sysmex^®^ Corporation of America). Clinical chemistry parameters were measured in 125 µl EDTA plasma samples with a Cobas Integra 400 plus machine (Roche Diagnostics).

### Euthanasia and necropsy

At the end of the study (day 14/15 or 28 in experiment 1, and day 21 in experiment 2) or upon reaching a humane endpoint, animals were euthanized by intramuscular injection of an anesthetic dose of Alfaxan/Ketamin (10 mg/kg, IM route) followed by an overdose of barbiturate (Pentobarbital, 70–100 mg/kg) injected into a vein in the groin. Complete necropsy was performed on all euthanized animals. Tissues were examined for gross pathology, frozen in liquid nitrogen, or fixed in 4% buffered formaldehyde for maximally 48 h. Formalin-fixed tissues were processed further by dehydration and embedding into paraffin.

### Oral and anal swabs

Oral and anal swabs were collected using FLOQSwabs® *516CS01* Ultra Minitip Flocked Swab with 100 mm Breakpoint (FLOQSwabs® COPAN Diagnostics Inc). Oral swabs were inserted into the oral cavity and the inside of each cheek was swabbed for a few seconds. The tip of each swab was placed into a 14 ml tube containing 2 ml of the transport medium (MEM, 0.5% BSA, 2.5 μg/ml Fungizone, 100 U/ml Penicillin, 100 μg/ml Streptomycin) and the applicator stick was broken off. Vials with medium and swabs were vortexed for 20 s. Supernatants were centrifuged for 5 min (750 × *g*) and stored in at least 2 aliquots at −80 °C. Anal swabs were placed in a transport medium and handled as described above. Possible debris was pelleted by centrifugation (750 × g, 5 min) and supernatants were collected and stored in at least two aliquots at −80 °C.

### Detection of viral RNA in plasma and organ suspensions

Frozen organ samples were thawed and subsequently homogenized using IKA Ultra Turrax Tube DT-20 in the presence of CIM supplemented with 1% a/a to generate 10% organ suspensions. The suspensions were transferred to 15 ml Falcon tubes and cell debris was removed by centrifugation for 15 min at 4952 × *g*. Organ suspensions (200 µl) or plasma samples (25 µl) obtained in Experiment 1 were added to 50 μl Proteinase K (5 μg/ml, Sigma). Next, 200 μl AL buffer (Qiagen), supplemented with 2 μl polyadenylic acid A (5 mg/ml, Sigma) was added, after which the samples were thoroughly mixed and incubated at 56 °C for 15 min. Subsequently, 250 μl 99% ethanol was added and RNA was isolated using the Qiagen RNeasy kit according to the manufacturer’s protocol. Organ suspensions (500 µl) or plasma samples (25 µl) obtained in experiment 2 were added to 2.5 ml NucliSENS easyMAG Lysis Buffer (Biomérieux, Marcy-l’Étoile, France), after which RNA was extracted using the NucliSENS easyMAG (Biomérieux) according to the manufacturer’s protocol. In both experiments, 5 μl of the RNA was used in an RT-qPCR using the LightCycler RNA Amplification Kit HybProbe (Roche, Almere, the Netherlands). Primers and probes were purchased from IDT. Forward primer: 5′-AAAGGAACAATGGACTCTGGTCA-3′, reverse primer: 5′-CACTTCTTACTACCATGTCCTCCAAT-3′; Probe: 5′-6FAM-AAAGCTTTGATATCTCTCAGTGCCCCAA-TMR-3′. Cycling conditions were as follows: 45 °C for 30 min, 95 °C for 5 min, 45 cycles of 5 s at 95 °C and 35 s at 57 °C, followed by cooling down to 30 °C.

### Virus isolation

Virus isolations were performed on all PCR-positive plasma samples and 10% organ suspensions with a threshold above 10^5^ RNA copies/ml as this has been previously shown to be a cut-off point below which no live virus can be detected^27^. Virus isolations were performed by serial dilution in complete CIM (supplemented with 5% FBS and 1% a/a) supplemented with 3.5 IU/ml heparin. Subsequently, the virus dilutions were incubated with BHK-21 cells. After 1.5 h incubation at RT, the inocula were replaced by fresh medium and after 5 days of culturing the cells at 37 °C and 5% CO_2_ cytopathic effects were scored.

### Virus neutralization test

Serum RVFV neutralizing antibodies were measured using a virus neutralization test (VNT)^28^. Briefly, serial dilutions (50 μl) of heat-inactivated sera (2 h, 56 °C) were incubated with 50 μl of RVFV-4s_eGFP_ (10^3.6^ TCID_50_/ml) for 2 h at RT. Subsequently, 20,000 BHK-21 cells (in 50 μl) were added to each well. Plates were incubated for 2 days at 37 °C and 5% CO_2_ and scored using an EVOS-FL microscope (Life Technologies). VNT_50_ titres were calculated using the Spearman–Kärber algorithm.

### Reporting summary

Further information on research design is available in the Nature Research Reporting Summary linked to this article.

## Supplementary information

Supplementary Info

REPORTING SUMMARY

## Data Availability

All data necessary to interpret, replicate, and build upon the methods or findings reported in the article are provided in this article.
