# Supplementary material for: Safety and immunogenicity of four-segmented Rift Valley fever virus in the common marmoset
Source: NPJ Vaccines. 2022 May 18;7:54. doi: 10.1038/s41541-022-00476-y (PMC9117246; doi:10.1038/s41541-022-00476-y)
Supplement: Supplementary file 1 — Supplementary Info [file 41541_2022_476_MOESM1_ESM.pdf]

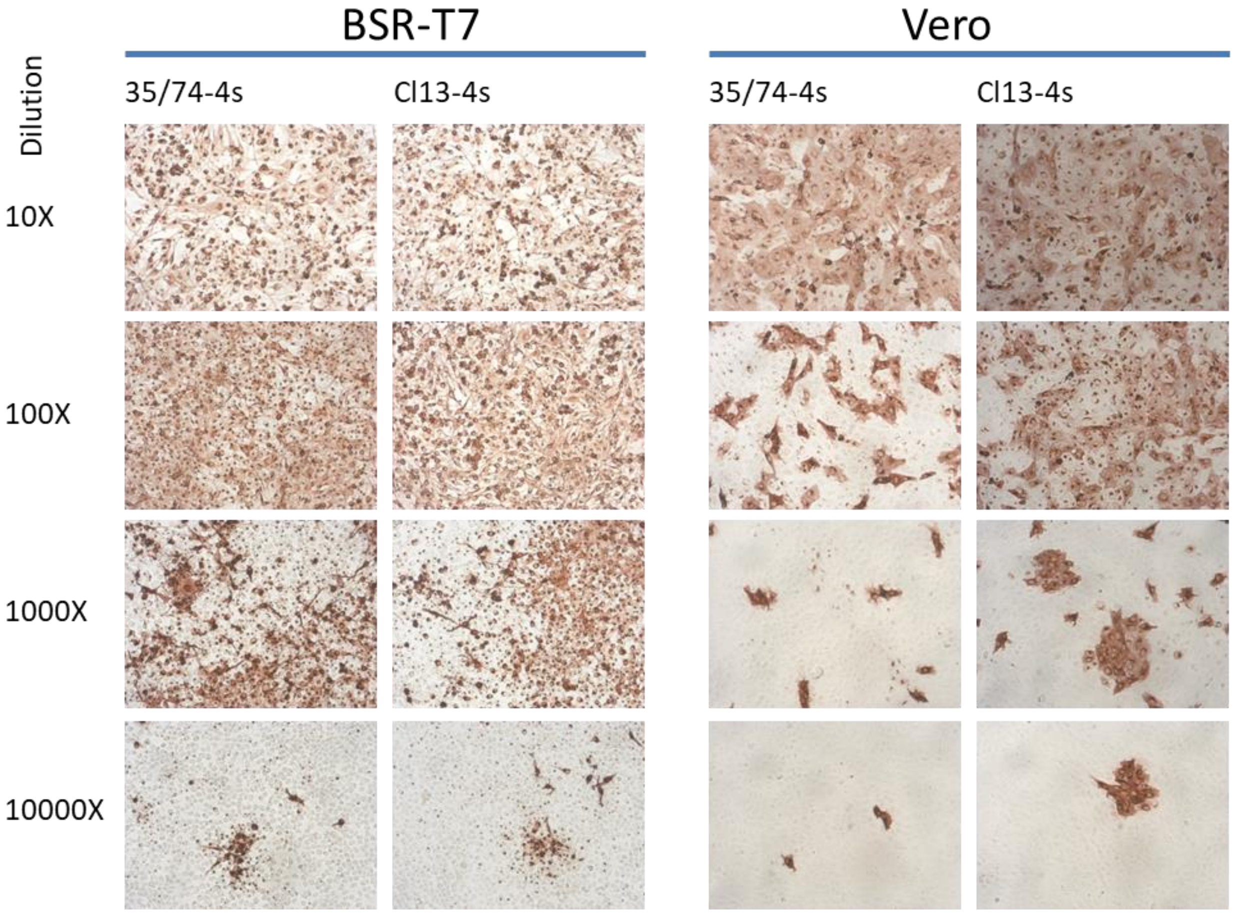

Supplementary Fig. 1A. Spread of vRVFV (35/74-4s) and hRVFV-4s (Cl13-4s) in BSR-T7 and Vero cells. Virus stocks of vRVFV (35/74-4s) and hRVFV-4s (Cl13-4s) produced on BSR-T7 cells were serially diluted (10 fold) and incubated on BSR-T7 and Vero cells for 2 days. Spread of the viruses was visualized by immunoperoxidase monolayer assay (IPMA) as described below. For vRVFV (35/74-4s) smaller plaques and less efficient spread was observed in Vero cells compared to hRVFV-4s (Cl13-4s).

Immunoperoxidase monolayer assay (IPMA)

Cells were fixed with 4% v/v paraformaldehyde for 15 min at RT and then washed with PBST20. For cell permeabilization, the fixed cells were incubated with PBS supplemented with 1% v/v Triton X-100 for 5 min at RT. After three washes with PBST20, plates were subsequently blocked with IPMA blocking buffer (5% v/v horse serum in PBS) and incubated for 1 hr at 37˚C. Primary antibody (4D4) was 1:50 diluted in blocking buffer (100 µL/well). Plates were incubated for 1 hr at 37˚C and then washed three times with PBST20. Subsequently, a HRP-conjugated rabbit anti-mouse IgG H+L secondary antibody (Dako P0260) in blocking buffer (1:500, 100 µL/well) was added. Plates were incubated for 1 hr at 37˚C and then washed three times with PBST20. For staining, 100

µL/well of a 0.2 mg/mL amino ethyl carbazole (AEC) solution in 500 mM substrate buffer (pH 5.0), 88 mM H2O2 was added as a substrate. Plates were incubated for 15–30 min at RT.

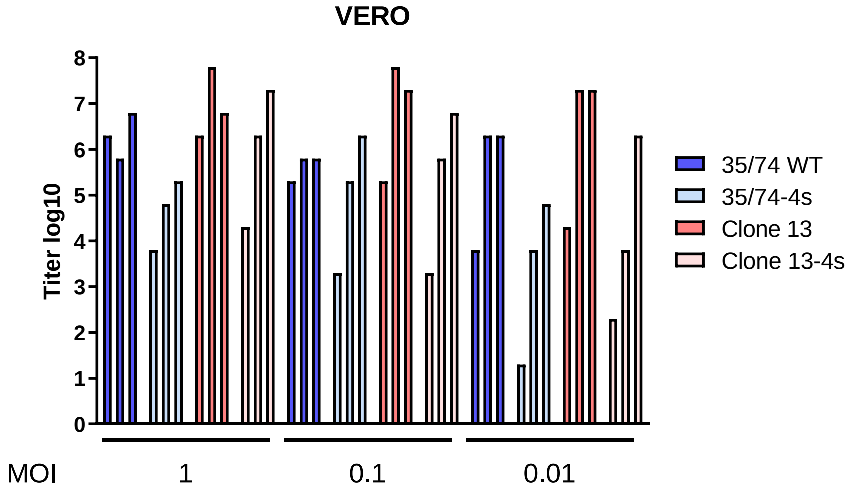

Supplementary Fig. 1B. RVFV-4s growth kinetics experiment in Vero cells. Growth of 35/74-4s and Cl13-4s was assessed on Vero cells following infections at a MOI of 1, 0.1 and 0.01. Supernatants were collected 1,2 and 3 days post infection and titrated on BSR-T7 (the first bar of each group corresponds to the titer at day 1, the second to the titer at day 2 and the third bar to the titer at day 3 post infection). Titers were calculated as the 50% tissue culture infective dose ($TCID_{50}$)/mL using the Spearman-Kärber method. Mean data of two biological replicates are presented for each bar.

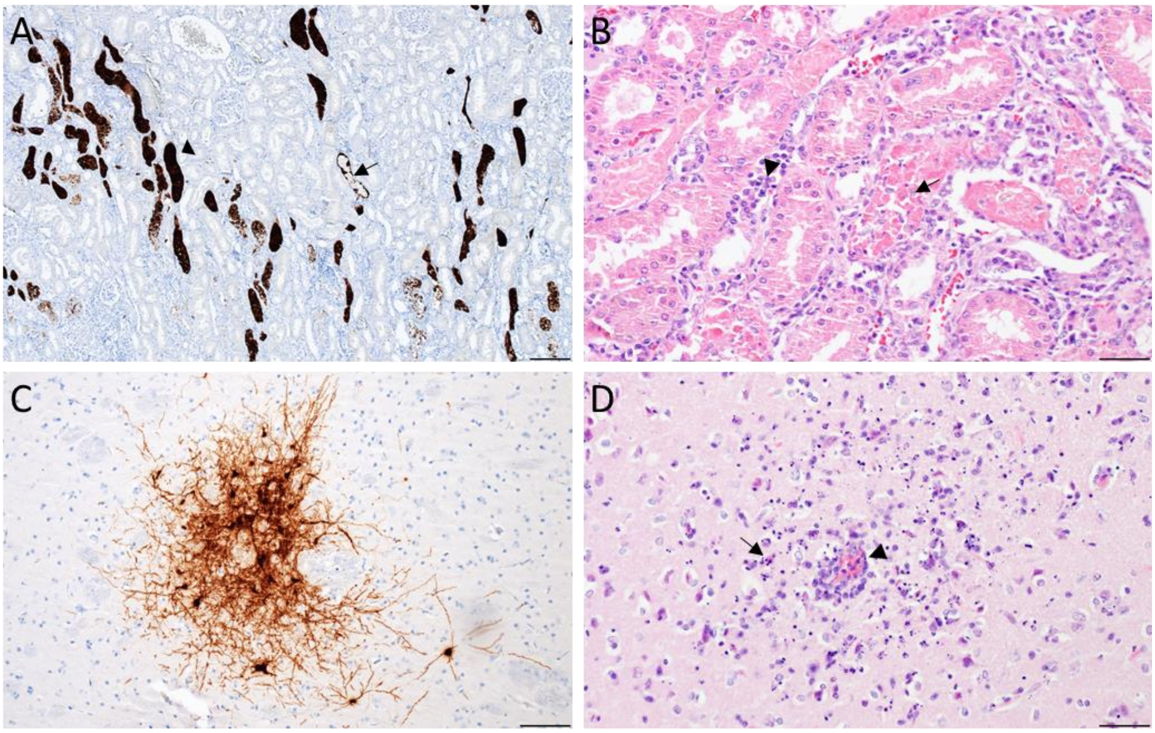

Supplementary Fig. 2. Panels **a** and **b**: Experiment 1, animal M12030, 15 days post inoculation with wild-type strain 35/74 at humane end-point. Kidney. (**a**) IHC: tubular epithelium (arrow) and casts within the tubular lumen (arrowhead) stain intensely for RVFV antigen, bar = 200 μm. (**b**) HE: tubulo-interstitial nephritis. Notice the degeneration and necrosis of the tubular epithelium and cellular debris in the lumen of the tubulus (arrow). Interstitial inflammatory reaction consisting mainly of mononuclear cells and few neutrophils (arrowhead), bar = 50 μm. Panels **c** and **d**: Experiment 2, animal M16040, 13 days post inoculation with wild type RVFV-74HB59 at humane end-point. Brain, thalamus. (**c**) IHC: focus of neurons and neuronal processes staining positively for RVFV antigen, bar = 100 μm. (**d**) HE: encephalitis with degeneration and necrosis of neurons (arrow) and nuclear debris scattered throughout the neuropil. Notice the perivascular cuffing (arrowhead) and infiltration of inflammatory cells (neutrophils and mononuclear cells), bar = 50 μm. Corresponding histology and immunohistochemistry methods are described below.

Histology and immunohistochemistry

Paraffin embedded tissues were cut into 4 μm sections, collected on silane-coated glass slides and dried for at least 48 h in a 37°C incubator. After deparaffinization and rehydration in graded alcohols, sections were stained routinely with haematoxylin and eosin (H&E) or immunostained for RVFV antigen. For immunostaining, epitopes were retrieved by autoclaving for 5 minutes at 121°C in pH 6 citrate buffer (Antigen unmasking solution, Vector Laboratories). RVFV Gn-specific monoclonal antibody 4-D4 was used as primary antibody. Specificity of the

immunostaining was confirmed with 2 other mAbs directed against the Gc and N protein of RVFV. HRP-conjugated anti-mouse IgG polymer (Invitrogen) was used as secondary antibody and DAB+ as substrate (Dako, Agilent) Sections were counterstained with haematoxylin.
