# Supplementary material for: Safety and immunogenicity of four-segmented Rift Valley fever virus in the common marmoset
Source: NPJ Vaccines. 2022 May 18;7:54. doi: 10.1038/s41541-022-00476-y (PMC9117246; doi:10.1038/s41541-022-00476-y)
Supplement: Supplementary file 2 — REPORTING SUMMARY [file 41541_2022_476_MOESM2_ESM.pdf]

nature portfolio

Corresponding author(s): Paul Wichgers Schreur

Last updated by author(s): Feb 7, 2022

Reporting Summary

Nature Portfolio wishes to improve the reproducibility of the work that we publish. This form provides structure for consistency and transparency in reporting. For further information on Nature Portfolio policies, see our Editorial Policies and the Editorial Policy Checklist.

Statistics

For all statistical analyses, confirm that the following items are present in the figure legend, table legend, main text, or Methods section.

n/a	Confirmed	
☐	☒	The exact sample size (n) for each experimental group/condition, given as a discrete number and unit of measurement
☒	☐	A statement on whether measurements were taken from distinct samples or whether the same sample was measured repeatedly
☐	☒	The statistical test(s) used AND whether they are one- or two-sided<br>*Only common tests should be described solely by name; describe more complex techniques in the Methods section.*
☒	☐	A description of all covariates tested
☒	☐	A description of any assumptions or corrections, such as tests of normality and adjustment for multiple comparisons
☐	☒	A full description of the statistical parameters including central tendency (e.g. means) or other basic estimates (e.g. regression coefficient) AND variation (e.g. standard deviation) or associated estimates of uncertainty (e.g. confidence intervals)
☒	☐	For null hypothesis testing, the test statistic (e.g. F, t, r) with confidence intervals, effect sizes, degrees of freedom and P value noted<br>*Give P values as exact values whenever suitable.*
☒	☐	For Bayesian analysis, information on the choice of priors and Markov chain Monte Carlo settings
☒	☐	For hierarchical and complex designs, identification of the appropriate level for tests and full reporting of outcomes
☒	☐	Estimates of effect sizes (e.g. Cohen's d, Pearson's r), indicating how they were calculated

Our web collection on statistics for biologists contains articles on many of the points above.

Software and code

Policy information about availability of computer code

Data collection	No specific software used
Data analysis	Data was analysed by Graphpad Prism 9.0

For manuscripts utilizing custom algorithms or software that are central to the research but not yet described in published literature, software must be made available to editors and reviewers. We strongly encourage code deposition in a community repository (e.g. GitHub). See the Nature Portfolio guidelines for submitting code & software for further information.

Data

Policy information about availability of data

All manuscripts must include a data availability statement. This statement should provide the following information, where applicable:
- Accession codes, unique identifiers, or web links for publicly available datasets
- A description of any restrictions on data availability
- For clinical datasets or third party data, please ensure that the statement adheres to our policy

All data necessary to interpret, replicate and build upon the methods or findings reported in the article are provided in this article.

Field-specific reporting

Please select the one below that is the best fit for your research. If you are not sure, read the appropriate sections before making your selection.

☒ Life sciences ☐ Behavioural & social sciences ☐ Ecological, evolutionary & environmental sciences

For a reference copy of the document with all sections, see nature.com/documents/nr-reporting-summary-flat.pdf

Life sciences study design

All studies must disclose on these points even when the disclosure is negative.

Sample size	No specific sample-size calculation was performed, though marmoset numbers/group were primarily chosen based on previous experiments with related viruses and anticipated variation.
Data exclusions	No data were excluded from the analyses.
Replication	Reproducibility was confirmed by performing two independent experiment as reported in the manuscript.
Randomization	Animals were sorted by age and gender and distributed over the groups.
Blinding	Blinding was not possible for this study, though objective read-out parameters were chosen as primary read-out parameters

Reporting for specific materials, systems and methods

We require information from authors about some types of materials, experimental systems and methods used in many studies. Here, indicate whether each material, system or method listed is relevant to your study. If you are not sure if a list item applies to your research, read the appropriate section before selecting a response.

Materials & experimental systems

n/a	Involved in the study	
☐	☒	Antibodies
☐	☒	Eukaryotic cell lines
☒	☐	Palaeontology and archaeology
☐	☒	Animals and other organisms
☒	☐	Human research participants
☒	☐	Clinical data
☒	☐	Dual use research of concern

Methods

n/a	Involved in the study	
☒	☐	ChIP-seq
☒	☐	Flow cytometry
☒	☐	MRI-based neuroimaging

Antibodies

Antibodies used	RVFV Gn-specific monoclonal antibody 4-D4 was used as primary antibody
Validation	For validation of this antibody we refer to: https://www.beiresources.org/Catalog/BEIMonoclonalAntibodies/NR-43190.aspx

Eukaryotic cell lines

Policy information about cell lines

Cell line source(s)	ATCC
Authentication	None of cell lines used were authenticated
Mycoplasma contamination	All cell lines were confirmed negative for mycoplasma
Commonly misidentified lines (See ICLAC register)	No commonly misidentified cell lines were used.

Animals and other organisms

Policy information about studies involving animals; ARRIVE guidelines recommended for reporting animal research

Laboratory animals	The experiments were carried out using purpose bred P2 adult common marmosets (Callithrix jacchus) of >350 gram. In experiment 1, 12 female animals (>2.5 years old) were enrolled whereas in experiment 2, 16 male and 8 female animals were used, also >2.5 years old.
Wild animals	The study did not involve wild animals.
Field-collected samples	The study did not involve samples collected from the field.
Ethics oversight	The study was reviewed and approved by the Dutch "Centrale Commissie Dierproeven" (AVD5020020174224) according to Dutch law, article 10a of the "Wet op de Dierproeven". The specific experiments were approved by BPRC's Animal Welfare Body (IvD).

Note that full information on the approval of the study protocol must also be provided in the manuscript.
